# Bidirectional fusion heterogeneous graph networks for semi-supervised Bitcoin transaction anomaly detection in dynamic transaction graphs

**DOI:** 10.1371/journal.pone.0351051

**Published:** 2026-06-08

**Authors:** Bo Xiao, Wei Yin

**Affiliations:** 1 School of Economics and Management, Southeast University, Nanjing, China; 2 The Laboratory of Philosophy and Social Sciences at Universities in Jiangsu Province-Fintech and Big Data Laboratory of Southeast University, Nanjing, China; Beijing Technology and Business University, CHINA

## Abstract

Detecting anomalies in the Bitcoin transaction network is critical for ensuring blockchain security and stability. The network’s heterogeneous structure and dynamic nature, coupled with scarce labeled anomalies, pose significant challenges for traditional graph-based methods. To address these, we propose Bidirectional Fusion Heterogeneous Graph Network (BF-HGN), a semi- dynamic supervised model for Bitcoin transaction anomaly detection task. BF-HGN designs multi-type feature embedding and alignment strategies to effectively unify features across heterogeneous transaction–address nodes. A bidirectional temporal fusion mechanism is proposed to capture long-range temporal dependencies that unidirectional models often miss. To alleviate class imbalance and limited annotations, a Class-balanced Classifier (CBC) combined with Adjacency Adaptation (AA) and Adaptive Feature Space Regulation (AFSR) losses is proposed to generate pseudo-anomalous nodes closely resembling real anomalies, improving discrimination boundaries. Experiments on the Elliptic++ dataset demonstrate that BF-HGN outperforms existing methods, achieving F1 scores of 0.6301 and 0.5784 for transaction and address nodes, respectively, establishing a new benchmark for Bitcoin transaction anomaly detection.

## 1 Introduction

Bitcoin, as a decentralized digital currency [1,2], has gained widespread attention by virtue of its transparency and security. Although Bitcoin transactions are observable on-chain, its anonymity [3] also facilitates illegal activities such as money laundering and dark-net transactions [4–7], threatening financial health and stability. Therefore, focusing on the anomaly detection task in Bitcoin transactions themselves has become a research focus, and its accurate identification can enhance network security and provide support for regulation. In this context, the data provided by exchange-based Know Your Customer (KYC) procedures is particularly important, as it enables the effective identification of target deposit addresses and the perpetrators behind them [8].

At the data processing stage, labeling abnormal samples in bitcoin transaction data relies on expertise and is costly, while labeling normal samples is relatively easy. Therefore, this study proposes a special semi-supervised learning framework that only a small number of normal samples are labeled. In addition, on-chain transaction data comprises both transaction and address nodes, which differ in their semantic meanings and topological roles. However, most previous studies [9–11]modeled transactions as a single type of node, thereby overlooking important cross-type relational information. Based on the aforementioned data characteristics, we integrate the Elliptic++ dataset [12] (Enriching heterogeneous information based on the Elliptic dataset [13]) to construct the first Bitcoin dynamic heterogeneous graph dataset.

In recent years, graph neural network (GNN) [14] has gradually become a research hotspot in Bitcoin transaction network analysis. Compared with traditional methods, GNNs are capable of efficient graph structure modeling, node embedding learning, and multi-level information integration, which can effectively handle network complexity and generate low-dimensional embedding representations. Commonly used techniques include graph convolutional network (GCN) [15] and graph autoencoder (GAE) [16]. Whereas, GCN-based methods have limitations in dealing with long distance dependencies, while GAE-based methods are suitable for unsupervised and semi-supervised tasks but are prone to overfitting problems when the data is extremely unbalanced. Specifically, Pareja et al. [17] proposed EvolveGCN, which extends GCN to learn representations of dynamic graphs. Zhao et al. [18] introduced GraphSMOTE, focusing solely on addressing class imbalance in transaction data. Liu et al. [19] developed EvolveGAN, emphasizing the capture of temporal evolution features in dynamic graphs. However, these methods have not fully addressed the combined challenges of transaction data heterogeneity, class imbalance, and temporal dynamics. To address the issues simultaneously, we propose a Bidirectional Fusion Heterogeneous Graph Network (BF-HGN), which consists of the multi-feature fusion-based feature extraction module and the Class-balanced Classifier (CC).

**Fig 1:**
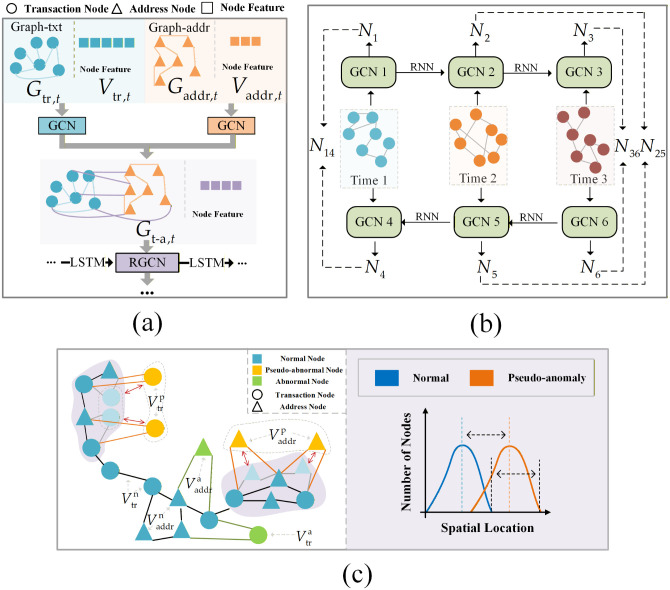
Overview of core innovative components. (a) Bidirectional temporal feature fusion: captures forward and reverse temporal dependencies via Bi-EvolveGCN and Bi-EvolveRGCN to mine long-range temporal associations; (b) Homogeneous-heterogeneous feature transformation: unifies feature dimensions of transaction and address nodes and constructs dynamic heterogeneous graphs; (c) Class-balanced Classifier: generates pseudo-anomalous nodes constrained by AA and AFSR losses to alleviate class imbalance in semi-supervised scenarios.

In the feature extraction stage, the heterogeneous graph contains node classes (Transactions and Addresses) with distinct feature dimensions and semantic spaces. To handle this heterogeneity, we experimented with several heterogeneous feature preprocessing strategies and determined the optimal one. As illustrated in Fig 1a, the proposed scheme first applies independent GCNs to the homogeneous subgraphs ($G_{\text{tr},t}$ and$G_{\text{addr},t}$) composed of transaction nodes and address nodes, respectively, to achieve feature-dimension unification and semantic alignment within each class. Based on the processed embeddings, a dynamic heterogeneous graph $G_{\text{t}-\text{a},t}$ is reconstructed, upon which RGCN-based [20] network is designed to extract cross-type, high-order relational features, thereby enhancing the model’s capability to represent heterogeneous information. Meanwhile, in dynamic modeling of the Bitcoin transaction network, temporal evolution is a crucial aspect. However, the basic RGCN cannot explicitly encode temporal dependencies between consecutive time steps. To address this limitation, we incorporate the idea of EvolveGCN [17], which couples Long Short-Term Memory (LSTM) [21] with graph convolutions, and propose an extended framework named EvolveRGCN. Nevertheless, conventional dynamic models typically employ unidirectional LSTM, which constrains their ability to learn long-range dependencies when the temporal span is large. To overcome this limitation, inspired by the design of Bidirectional LSTM (Bi-LSTM) [22] in dynamic signal anomaly detection [23,24], we further propose two bidirectional variants—Bi-EvolveGCN and Bi-EvolveRGCN—for feature extraction in our task. They fuse node features from forward and reverse temporal iterations to enhance the model’s ability to model long-term dynamic transactions. As demonstrated in Fig 1b, taking the subgraph at time point 2 as an example, the fused feature $N_{\text{25}}$ is generated by aggregating the forward feature at time point 2 and the reverse feature $N_{\text{2}}$ at time point 2 and the reverse feature $N_{\text{5}}$ at time point 5. This mechanism effectively enhances the model’s ability to mine associated features between nodes over a long-time span.

In the classification stage, to address the problem of severe class imbalance caused by unlabeled abnormal samples, we propose the Class-balanced Classifier (CC). Its aim is to generate pseudo-abnormal nodes that have similar characteristics to real hard abnormal nodes. The generation process comprehensively considers two core characteristics of real abnormal nodes. One characteristic stems from the differences in the adjacency relationships between real abnormal nodes and normal nodes. We designed the Adjacency Adaptation (AA) loss function to adjust the adjacency relationships of pseudo-abnormal nodes. Specifically, as shown on the left side of Fig 1c, the pseudo-abnormal transaction nodes $\overset{\text{p}}{\underset{\text{tr}}{V}}$ and address node set $\overset{\text{p}}{\underset{\text{addr}}{V}}$ are generated through transformation by referring to the adjacency relationships of the real abnormal node sets $\overset{\text{n}}{\underset{\text{tr}}{V}}$ and $\overset{\text{n}}{\underset{\text{addr}}{V}}$ and adjusting the adjacency relationships of some normal node sets $\overset{\text{a}}{\underset{\text{tr}}{V}}$ and $\overset{\text{a}}{\underset{\text{addr}}{V}}$. The second characteristic, as shown on the right side of Fig 1c, targets the problem that the core of the task lies in the high similarity between some abnormal nodes and normal nodes in terms of features, which makes detection difficult. We propose Adaptive Feature Space Regulation (AFSR) loss function, which optimizes the model’s ability to recognize hard abnormal situations by controlling the distribution of the pseudo-abnormal and the normal node set in the feature space.In summary, the main innovations of this paper are as follows:

1. The task of “semi-supervised bitcoin anomaly detection for dynamic heterogeneous graphs” is defined for the first time, and the Bidirectional Fusion Heterogeneous Graph Network (BF-HGN) is designed, which provides a preliminary modeling framework for this field.
2. To address the dynamic and heterogeneous nature of Bitcoin transaction data, we propose EvolveRGCN—a relational graph convolutional network designed for feature extraction in dynamic settings. Building upon EvolveGCN, we further develop a progressive feature learning strategy that transitions from homogeneous to heterogeneous representations, enabling deeper semantic mining of transaction structures. To better capture under-explored temporal dependencies, we introduce Bi-EvolveGCN and Bi-EvolveRGCN, which incorporate bidirectional feature fusion. By integrating node representations from both forward and reverse temporal directions, these models effectively capture complex dependencies across temporal subgraphs.
3. To tackle the class imbalance problem caused by unlabeled abnormal samples in transactions, we design CC module, aiming to generate pseudo-abnormal nodes whose feature structures are similar to those of real hard abnormal nodes. This process is constrained by the proposed dual loss functions: the AA loss function constrains its relationship with adjacent nodes, and the AFSR loss function constrains its feature space distribution.

## 2 Related work

### 2.1 Bitcoin transaction anomaly detection

Bitcoin has gained widespread application in the financial transaction sector due to its anonymity and immutability. Yet, these characteristics also contribute to more illicit transactions thought Bitcoin. Thus, the Bitcoin transaction anomaly detection task emerges as the times require. Its core lies in analyzing blockchain transaction records to identify anomalies, a process that can be conducted using elements such as the graph structure characteristics of the transaction network, node attributes, and dynamic patterns [25]. Current mainstream detection methods can be broadly categorized into two types: the first is feature engineering-based methods, which extract features such as transaction frequency, amount, and node connectivity, and utilize traditional machine learning algorithms like support vector machines (SVM) [26] and random forests (RF) [27] for detection. Such methods do not take into account the relationships between transactions; The second category involves deep learning-based methods [28], which construct dynamic graph neural networks (DGNNs) or self-supervised learning models to learn latent features, thereby enhancing detection accuracy and robustness. It can effectively capture time-series and node interaction information [19,29]. Although related research has made progress, challenges such as scarce labeled data and the strong concealment of malicious behavior remain. In this context, leveraging unsupervised and semi-supervised learning techniques to enhance detection capabilities has become an important future research direction [30].

### 2.2 Dynamic graph model for Bitcoin transaction anomaly detection

With the development of GNNs, the dynamic Graph Anomaly Detection (DGAD) methods [31,32] have also demonstrated excellent performance in the task of Bitcoin transaction anomaly detection. This section mainly discusses the three DGAD settings: supervised, unsupervised, and semi-supervised.

Supervised learning constructs models using labeled data to detect anomalies in new dynamic graphs. This method achieves high accuracy and effectively learns the differences between normal and anomalous patterns. It can be primarily divided into two categories. The first category is classification-based methods, such as SVM and decision trees. Taking the dynamic social network as an example, such methods perform anomaly detection solely by inputting node features at different time points; the second is deep learning methods, such as using CNN and RNN to process dynamic graph sequence data, extract features, and apply them to classification tasks [33,34].

Unsupervised learning does not require labeled data; it identifies anomalies based on the distribution and structural characteristics of the data, if normal data follows specific patterns while anomalous data deviates from these patterns. This approach is suitable for scenarios where labeled data is difficult to obtain. Commonly used methods include density-based algorithms (e.g., LOF [35] identifies anomalies by calculating local density), clustering methods (where normal data forms tight clusters and anomalous points deviate from the cluster center), and reconstruction-based graph autoencoders (GAE reconstructs graph structures, such as DOMINANT [36] using GCN to implement GAE’s graph structure reconstruction, while AnomalyDAE [37] enhances structural reconstruction performance). Additionally, some researchers have proposed the TAM [38] method to conduct in-depth exploration of the relationships between nodes and subgraphs, but there is still room for improvement in its utilization of labeled information.

Semi-supervised learning lies between supervised and unsupervised learning, utilizing a small amount of labeled data and a large amount of unlabeled data to reduce the demand for labeling while enhancing the model’s generalization ability. Current methods can be divided into traditional classification algorithms (such as semi-supervised SVM, which constructs an initial classifier and iteratively updates it) and deep learning algorithms based on GCN. Although one-class classification [39–41] is widely applied in visual data, it is rarely seen in the node-level anomaly detection task for dynamic graphs. A common requirement for this type of task is that both normal nodes and abnormal nodes are labeled, which has high requirements in terms of cost. By comparison, the semi-supervised method of one-class classification is more practical in the Bitcoin transaction anomaly detection task.

### 2.3 Dynamic heterogeneous graph model for Bitcoin transaction anomaly detection

The key distinction between GNNs and other neural networks lies in their adherence to a message-passing mechanism [42], whereby each node aggregates information from its neighboring nodes. Heterogeneous GNNs further consider the heterogeneity of the graph, independently distinguishing between various types of nodes and edges. Dynamic heterogeneous graph neural networks (DHGNNs) further explore time-based information in dynamic graphs. In real-world scenarios, such as the Elliptic++ dataset [12], Bitcoin transactions at each time step are formed through interactions between transaction nodes and address nodes. Essentially, this type of data can be regarded as a form of dynamic heterogeneous graph data. Zhang et al. proposed DHGAS, which encodes temporal information [43] before performing heterogeneous message passing; others employed sequence-based models to aggregate information from different time slices [19]. Additionally, in semi-supervised heterogeneous graph anomaly detection tasks, researchers often adopt methods based on Generative Adversarial Networks (GANs) [44,45], which leverage adversarial training with a small amount of label information to enhance the model’s ability to perceive anomalies. Along this line of research, Nair et al. proposed a data-driven risk analytics framework using semi-supervised heterogeneous graph modeling for blockchain transaction fraud [46]. Similarly, Santos et al. studied adaptive graph neural analytics for cryptocurrency anomaly detection under limited labeled data conditions [47]. Finally, recent advances have presented new ideas for the task of heterogeneous and temporal feature fusion. H2CAN [48] uses heterogeneous hypergraph attention to model high-order cross-modal interactions and adopts counterfactual learning to reduce bias for multimodal sentiment analysis. STEAM [49] mines structural-temporal features via motif-augmented hypergraphs and fine-grained temporal autoencoder to detect motif-level anomalies in dynamic graphs.

## 3 Method

Facing the semi-supervised, dynamic, and heterogeneous characteristics of real-world Bitcoin transaction data, we propose the Bidirectional Fusion Heterogeneous Graph Network (BF-HGN) for anomaly detection tasks. BF-HGN encompasses two core modules: the Multi-type Feature Fusion Extractor (MFFE) and the Class-balanced Classifier (CC). During the feature extraction phase, MFFE enriches the heterogeneous information between transaction and address nodes on the basis of extracted basic features. It employs a bidirectional temporal fusion mechanism to embed temporal information, enabling in-depth mining of cross-time subgraph dependencies. This module includes the Bi-EvolveRGCN and Bi-EvolveGCN core sub-networks, the technical details of which are presented in Section 3.1. In the classification phase, CC is designed to generate pseudo-anomalous nodes similar to real hard anomalous nodes, mitigating the class imbalance issue. The specific implementation is detailed in Section 3.2. Section 3.3 elaborates on the model’s loss function system, where the AA loss function and the AFSR loss function play crucial guiding roles in the generation of the pseudo-anomalous node set.

According to the dynamic characteristics of the data in this task, it is divided into subgraphs at consecutive T time points. The transaction graph and the address graph at a certain time point t are denoted as $G_{\text{tr},t}=(X_{\text{tr},t},A_{\text{tr},t})$ and $G_{\text{addr},t}=(X_{\text{addr},t},A_{\text{addr},t})$ respectively. Taking $G_{\text{tr},t}$ as an example, $X_{\text{tr},t}\in R^{N\times d}$ represents the node features of $G_{\text{tr},t}$, where d is the dimension of the features; $A_{\text{tr},t}\in R^{N\times N}$ is the adjacency matrix for the edge relationships of $G_{\text{tr},t}$ $(A_{\text{tr},t}[i,j]=\text{1}$ if there is an edge between the source node i and the target node j, and 0 otherwise.

### 3.1 Multi-class feature fusion extractor

For the dynamic graph scenario, the EvolveGCN can only capture the dependency relationships of dynamic subgraphs in the forward time direction and is insufficient in mining the dependency relationships in the reverse time direction. To address this, we propose a dual-time-direction feature fusion mechanism and constructs the Bi-EvolveGCN network to achieve bidirectional feature aggregation of dynamic subgraphs. Additionally, we design the Bi-EvolveRGCN feature extraction network suitable for dynamic heterogeneous graphs based on LSTM and RGCN. Moreover, the preprocessing step for heterogeneous features is crucial for the model to subsequently learn the complex heterogeneous relationships between nodes, directly influencing the extraction efficiency of key information and the ability to identify abnormal situations.

#### 3.1.1 Bi-EvolveGCN.

**Fig 2:**
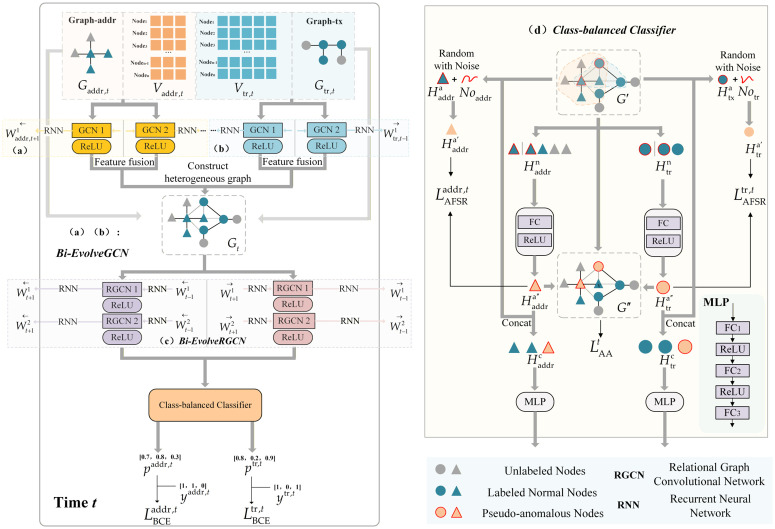
Shows the framework of BF-HGN. First, the address graph ${𝐆}_{\text{addr},t}$ and transaction graph ${𝐆}_{\text{tr},t}$ are processed by (a) and (b) Bi-EvolveGCN respectively to unify the dimensions of all nodes, so as to construct the heterogeneous graph ${𝐆}_{t}$. Then, ${𝐆}_{t}$ goes through (c) Bi-EvolveRGCN for feature extraction to obtain the sub-graph ${𝐆}^{\prime }$. Finally, ${𝐆}^{\prime }$ is input into (d) CC to achieve node classification.

Taking time point t as an example, (a) and (b) in Fig 2 are respectively implemented based on two EvolveGCN networks, and the core difference between two lies in the opposite time directions of parameter update. By fusing the feature information in the forward and reverse directions, the Bi-EvolveGCN is finally constructed, which integrates the node features extracted by both.

Next, this section will take the Bi-EvolveGCN at the l-th layer as an example for introduction. Before it, we will first explain the meanings of the common parameters in the subsequent formulas: $\overset{\leftarrow }{\overset{l}{\underset{\text{tr},t}{W}}}$ and $\overset{\to }{\overset{l}{\underset{\text{tr},t}{W}}}$ are the learnable parameters of the set of the transaction node set updated in the forward and backward time directions at the time t, respectively. Firstly, we focus on the updating process of $\overset{\leftarrow }{\overset{l}{\underset{\text{tr},t}{W}}}$ and $\overset{\to }{\overset{l}{\underset{\text{tr},t}{W}}}$, where the update formula for $\overset{\leftarrow }{\overset{l}{\underset{\text{tr},t}{W}}}$ is:


$$\begin{array}{c} \overset{\leftarrow }{\overset{l}{\underset{\text{tr},t}{W}}}=\text{LSTM}\left(\overset{\leftarrow }{\overset{l}{\underset{\text{tr},t-\text{1}}{W}}}\right) \end{array}$$ (1)

LSTM, as a special type of RNN, can effectively capture the dependency relationships in time series. The forward learnable weight parameter $\overset{\leftarrow }{\overset{l}{\underset{\text{tr},t-\text{1}}{W}}}$ at time t−1 is input into the LSTM unit. Correspondingly, the update formula for $\overset{\to }{\overset{l}{\underset{\text{tr},t}{W}}}$ is:


$$\begin{array}{c} \overset{\to }{\overset{l}{\underset{\text{tr},t}{W}}}=\text{LSTM}\left(\overset{\to }{\overset{l}{\underset{\text{tr},t+\text{1}}{W}}}\right) \end{array}$$ (2)

where $\overset{\to }{\overset{l}{\underset{\text{tr},t}{W}}}$ at time t is updated by $\overset{\to }{\overset{l}{\underset{\text{tr},t+\text{1}}{W}}}$ at time t+1. Through the forward and backward time-directed LSTM updating mechanism, the model is able to better fit the temporal change characteristics of the dynamic graph.

Subsequently, further node feature updating operations in the forward and backward time directions are performed based on $\overset{\leftarrow }{\overset{l}{\underset{\text{tr},t}{W}}}$. The update formula for the forward node feature $\overset{\leftarrow }{\overset{l+\text{1}}{\underset{\text{tr},t}{X}}}$ at layer l+1 is as follows:


$$\begin{array}{c} \overset{\leftarrow }{\overset{l+\text{1}}{\underset{\text{tr},t}{X}}}=\delta \left(A_{\text{tr},t}\overset{\leftarrow }{\overset{l}{\underset{\text{tr},t}{X}}}\overset{\leftarrow }{\overset{l}{\underset{\text{tr},t}{W}}}+\overset{\leftarrow }{\overset{l}{\underset{\text{tr},t}{b}}}\right) \end{array}$$ (3)

where δ() is a ReLU [50] linear transformation, and nonlinear factors can be introduced to enhance the model representation. $A_{\text{tr},t}$ is the transaction class node adjacency matrix at time t, describing node connectivity. $\overset{\leftarrow }{\overset{l}{\underset{\text{tr},t}{X}}}$ is the lth layer forward transaction class node feature at time t, and $\overset{\leftarrow }{\overset{l}{\underset{\text{tr},t}{b}}}$ is the corresponding intercept. Correspondingly, the update formula for the reverse node feature $\overset{\to }{\overset{l}{\underset{\text{tr},t}{W}}}$ based on the output of $\overset{\to }{\overset{l+\text{1}}{\underset{\text{tr},t}{X}}}$ is:


$$\begin{array}{c} \overset{\to }{\overset{l+\text{1}}{\underset{tr,t}{X}}}=\delta (A_{tr,t}\overset{\to }{\overset{l}{\underset{tr,t}{X}}}\overset{\to }{\overset{l}{\underset{tr,t}{W}}}+\overset{\to }{\overset{l}{\underset{tr,t}{b}}}) \end{array}$$ (4)

Finally, if the total number of layers of Bi-EvolveGCN is l, it is necessary to perform the fusion operation of the node features $\overset{\leftarrow }{\overset{l+\text{1}}{\underset{\text{tr},t}{X}}}$ and $\overset{\to }{\overset{l+\text{1}}{\underset{\text{tr},t}{X}}}$, as given in equation:


$$\begin{array}{c} X_{\text{tr},t}=\text{Concat}((\overset{\leftarrow }{\overset{l+\text{1}}{\underset{\text{tr},t}{X}}},\overset{\to }{\overset{l+\text{1}}{\underset{\text{tr},t}{X}}}),\text{1}) \end{array}$$ (5)

The above operation is to splice $\overset{\leftarrow }{\overset{l+\text{1}}{\underset{\text{tr},t}{X}}}$ with $\overset{\to }{\overset{l+\text{1}}{\underset{\text{tr},t}{X}}}$ in the 1st dimension to obtain the transaction class node feature $X_{\text{tr},t}$ extracted by Bi-EvolveGCN. Similarly, the address node class feature $X_{\text{addr},t}$ can be obtained by the same method.

#### 3.1.2 Bi-EvolveRGCN.

Building on the design concept of Bi-EvolveGCN, (c) in Fig 2 is based on RGCN and also adopts the strategy of bidirectional time-dependent learnable parameter update, thus designing Bi-EvolveRGCN suitable for dynamic heterogeneous graphs. The core difference between RGCN and GCN is that the former achieves the heterogeneous graph feature extraction by aggregating the neighboring nodes of multi-class edges separately, while GCN does not design a special mechanism for aggregating the neighboring nodes of multi-class edges.

Next, this section starts with the introduction based on the lth layer Bi-EvolveRGCN. Before it, the meanings of common parameters are first interpreted: $\overset{r}{\underset{i}{N}}$ represents the set of nodes adjacent to node i with an adjacency relationship r∈R(R is the set of edge classes); $\overset{\leftarrow }{\overset{l+\text{1}}{\underset{i,t}{h}}}$ and $\overset{\to }{\overset{l+\text{1}}{\underset{i,t}{h}}}$ are the features of node i updated in the forward and backward time direction, respectively, by the l-th layer of RGCN at time t. Among them, the update formula for $\overset{\leftarrow }{\overset{l+\text{1}}{\underset{i,t}{h}}}$ is as follows:


$$\begin{array}{c} \overset{\leftarrow }{\overset{l+\text{1}}{\underset{i,t}{h}}}=\sum_{r\in R}\sum_{j\in \overset{r}{\underset{i}{N}}}\frac{\text{1}}{c_{i,r}}\delta (\overset{\leftarrow }{\overset{l}{\underset{r,t}{W}}}\overset{\leftarrow }{\overset{l}{\underset{j,t}{h}}}+\overset{\leftarrow }{\overset{l}{\underset{r,t}{b}}}) \end{array}$$ (6)

where δ() represents the ReLU linear transformation; $\overset{\leftarrow }{\overset{l}{\underset{r,t}{b}}}$ is the learnable bias used to update nodes under the adjacency relationship r. $c_{i,r}$ is the number of nodes of node i under the adjacency r, which is used to normalize the process of feature update. $\overset{\leftarrow }{\overset{l}{\underset{r,t}{W}}}$ is updated from $\overset{\leftarrow }{\overset{l}{\underset{r,t-\text{1}}{W}}}$ and is used to update the matrix weight parameter of the node set under the adjacency relationship r∈R for the target node in the forward time direction. Accordingly, $\overset{\to }{\overset{l+\text{1}}{\underset{i,t}{h}}}$ is updated by the following equation:


$$\begin{array}{c} \overset{\to }{\overset{l+\text{1}}{\underset{i,t}{h}}}=\sum_{r\in R}\sum_{j\in \overset{r}{\underset{i}{N}}}\frac{\text{1}}{c_{i,r}}\delta (\overset{\to }{\overset{l}{\underset{r,t}{W}}}\overset{\to }{\overset{l}{\underset{j,t}{h}}}+\overset{\to }{\overset{l}{\underset{r,t}{b}}}) \end{array}$$ (7)

where $\overset{\to }{\overset{l}{\underset{r,t}{W}}}$ is the matrix weight parameter updated from $\overset{\to }{\overset{l}{\underset{r,t+\text{1}}{W}}}$.

#### 3.1.3 Feature transformation method.

**Fig 3:**
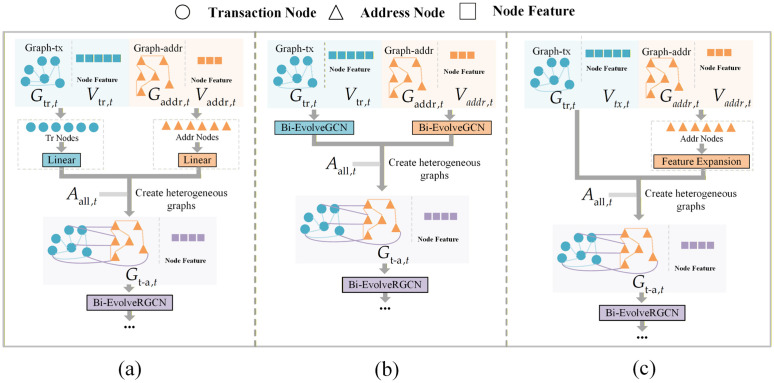
Schematic diagram of scheme comparison. (a) shows the scheme of node set feature transformation using FC; (b) depicts the process of transforming graph node set features through GNN-based modeling; and (c) shows the scheme of carrying out feature expansion for node sets with less feature dimensions.

To screen the optimal feature transformation [51] methods, we compare and analyzes various schemes through Fig 3, including the use of fully connected layers (FC), GCN, etc. for node-set feature transformation, as well as the expansion operation for low-dimensional feature node sets. The influence of different fusion methods on the model effect is further explored later in Section 4.4.

### 3.2 Fully connected layer feature transformation methods

As shown in Fig 3a, we adopt two FC layers to transform the features of different nodes respectively, so that the feature dimensions of different nodes are consistent. The specific realization of the formula is:


$$\begin{array}{c} G_{t}=\text{GHG}((\text{Linear}(V_{\text{tr},t}),\text{Linear}(V_{\text{addr},t})),A_{t}) \end{array}$$ (8)

where GHG((x,y),z) is defined as the process where the two types of node sets x and y are fused with all adjacent edges y to generate a heterogeneous graph $G_{t}$ which is then input into the multi-layer Bi-EvolveRGCN for feature extraction.

### 3.3 GCN feature transformation methods

As shown in Fig 3b, we employ two Bi-EvolveGCN to pre-adjust the homogeneous graph data respectively. The major difference between this method and the first method lies in that it fully considers the connection relationship between homogeneous nodes. The specific implementation is as follows:


$$\begin{array}{c} G_{t}=\text{GHG}((\text{Bi}-\text{EGCN}(G_{\text{tr},t}),\text{Bi}-\text{EGCN}(G_{\text{addr},t})),A_{t}) \end{array}$$ (9)

where $G_{\text{tr},t}$ and $G_{\text{addr},t}$ denote the transaction homogeneous graph and address homogeneous graph, respectively, and $G_{t}$ is the heterogeneous graph. Specifically, $G_{\text{tr},t}$ and $G_{\text{addr},t}$ are the fundamental homogeneous components, and $G_{t}$ is formed by integrating these two homogeneous subgraphs.

### 3.4 Random forest feature expansion methods

**Fig 4:**
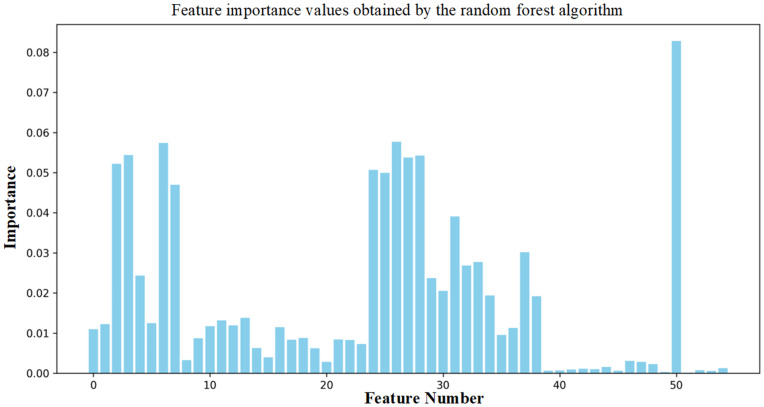
Shows the importance values of each feature of the address node.


Fig 3c adopts a feature expansion method to augment the features of the node class with low feature dimensions (the address node class) to achieve the matching of data dimensions among different node classes. Given the advantages of the Random Forest (RF) algorithm in feature importance assessment, we introduce it for pre-training to obtain the importance value of each feature. For this purpose, we obtain the feature importance sequence Inp (the sum of values being 1) as shown in Fig 4. Each feature is expanded based on this ratio to achieve the unification of feature dimensions for different node classes. The specific formula is as follows:

$$\begin{array}{c} G_{t}=\text{GHG}((\text{expand}(V_{\text{addr},t},\text{Inp}),V_{\text{tr},t}),A_{t}) \end{array}$$ (10)

where expand(x,y) is the operation of feature expansion on the node set x based on the importance array y.

Based on the comparison experiments in Section 4.4, the feature extraction scheme fusing Bi-EvolveGCN and Bi-EvolveRGCN is selected. This scheme effectively realizes multi-category node feature conversion and extraction with excellent performance.

### 3.5 Class-balanced classifier

In the classification stage, we design an innovative Class-balanced Classifier to accomplish the effective classification task of multi-class nodes. As shown in Fig 2d, $G^{\prime }=(H_{\text{x}}\in R^{N\times d},A\in R^{N\times N})$ is the sub-graph after the feature extraction stage at time t. Here, $H_{\text{x}}$ (x∈{tr,addr}) and E are the node set and the adjacency matrix respectively, N is the total number of nodes in the graph, and d is the dimension of the output node features.

First, we randomly select the sets of labeled normal nodes with a proportion of δ (node ID sets $I_{\text{a}}$, total number $N_{\text{a}})$ as the node set to be converted, denoted as $\overset{\text{a}}{\underset{\text{x}}{H}}=H_{\text{x}}[\overset{\text{a}}{\underset{\text{x}}{I}},:]$. aims to control the proportion of generated abnormal nodes, so as to help BF-HGN learn the feature differences between normal and abnormal nodes. Next, $\overset{\text{a}}{\underset{\text{x}}{H}}$ embeds the corresponding neighbor nodes to obtain the initial set of pseudo-abnormal nodes, and the formula is as follows:


$$\begin{array}{c} \overset{\text{n}}{\underset{\text{x}}{H}}=A_{x}[\overset{\text{a}}{\underset{\text{x}}{I}},:]\times H_{\text{x}}[\overset{\text{a}}{\underset{\text{x}}{I}},:] \end{array}$$ (11)

where $A_{\text{x}}\in R^{N_{\text{x}}\times N_{\text{x}}}$ is the adjacency matrix of $H_{\text{x}}$ and $\overset{\text{n}}{\underset{\text{x}}{H}}\in R^{N_{\text{x}}\times d_{\text{x}}}$ represents the neighbor embedding features of the initial abnormal nodes. Meanwhile, the noise matrix ${No}_{\text{x}}$ that obeys a specific normal distribution (mean μ, variance$\sigma^{\text{2}}$) and has a dimension consistent with the pseudo anomalous node feature $\overset{\text{a}}{\underset{\text{x}}{H}}$ is pre-generated and added to $H_{\text{x}}[\overset{\text{a}}{\underset{\text{x}}{I}},:]$:


$$\begin{array}{c} {No}_{\text{x}}=\text{Randn}(\overset{\text{a}}{\underset{\text{x}}{H}}.\text{size})\times \sigma^{\text{2}}+\mu ) \end{array}$$ (12)

$$\begin{array}{c} \overset{{\text{a}}^{,}}{\underset{\text{x}}{H}}=\overset{\text{a}}{\underset{\text{x}}{H}}+{No}_{\text{x}} \end{array}$$ (13)

where $\text{Randn}(H_{\text{x},t}[\overset{\text{a}}{\underset{\text{x},t}{I}},:].\text{size})$denotes the generation of a normally distributed random number matrix with the same dimension as ${\overset{\text{a}}{\underset{\text{x}}{H}}=H}_{\text{x}}[\overset{\text{a}}{\underset{\text{x}}{I}},:]$. $\overset{\text{o}}{\underset{\text{x},t}{H}}$ is random and cannot simulate real abnormal nodes, so it cannot be directly defined as a pseudo – abnormal node. The purpose of adding noise to it is to form a symmetry in the feature space with the normal node set, which is consistent with the characteristics of hard abnormal nodes and can guide the generation of pseudo-abnormal nodes to be biased towards hard abnormal nodes.

Subsequently, the FC and ReLU activation layers are sequentially introduced to process $\overset{\text{n}}{\underset{\text{x}}{H}}$ with the following equations:


$$\begin{array}{c} \overset{{\text{a}}^{\prime \prime }}{\underset{\text{x}}{H}}=\text{ReLU}(\text{FC}(\overset{\text{n}}{\underset{\text{x}}{H}})) \end{array}$$ (14)

Then, the normal node features $H_{\text{x}}[\overset{\text{n}}{\underset{\text{x}}{I}},:]$ are sequentially connected to the features of $\overset{{\text{a}}^{\prime }}{\underset{\text{x},t}{H}}$ with the following formula:


$$\begin{array}{c} \overset{\text{c}}{\underset{\text{x}}{H}}=\text{Concat}((H_{\text{x}}[\overset{\text{n}}{\underset{\text{x}}{I}},:],\overset{{\text{a}}^{\prime \prime }}{\underset{\text{x},}{H}}),\text{0}) \end{array}$$ (15)

where $\overset{\text{n}}{\underset{\text{x}}{I}}\in R^{\overset{\text{n}}{\underset{\text{x}}{N}}}$ is the ID set of labeled normal nodes. Concat((a,b),c) denotes splicing the a node set and the b node set in the cth dimension. Meanwhile, $\overset{{\text{a}}^{\prime \prime }}{\underset{\text{x}}{H}}$ is used to replace the pseudo anomalous node set in $H_{\text{x}}$, and the formula is as follows:


$$\begin{array}{c} G^{\prime \prime }=\text{Repalace}((G^{\prime },\overset{{\text{a}}^{\prime \prime }}{\underset{\text{x}}{H}}),\overset{\text{a}}{\underset{\text{x}}{I}}) \end{array}$$ (16)

where Repalace((a,b),c) means replacing the node features of a with the node features of b by taking c as the reference number for corresponding nodes. Finally, a multilayer perceptron (MLP) [52] consisting of three FC and two ReLU activation layers is introduced to perform the node classification task with the following formula:


$$\begin{array}{c} P_{\text{x},t}=\text{MLP}(\overset{\text{c}}{\underset{\text{x}}{H}}) \end{array}$$ (17)

where MLP() is a multilayer perceptual machine method with $P_{\text{x},t}\in R^{\overset{\text{a}}{\underset{\text{x}}{N}}+\overset{\text{n}}{\underset{\text{x}}{N}}}$.

### 3.6 Loss function

To generate abnormal nodes that are more conducive to model learning, we propose an AA loss function and AFSR loss function to constrain the generation of pseudo-abnormal nodes. Meanwhile, the Binary Cross-entropy (BCE) loss is set as the basic loss function.

#### 3.6.1 Loss function for the degree of neighbor aggregation.

To incorporate the graph structure prior of abnormal nodes into the generation process of outlier nodes, BF-HGN leverages the differential characteristic of the adjacency aggregation degree, forcing the aggregation degree of pseudo-abnormal nodes towards their neighbors to be lower than that of normal nodes. In$G^{\prime \prime }=(E,A)$, E represents the features of all nodes. First, the function needs to calculate the normalized similarity matrix $S_{ij}$ between the target node and its various types of neighbor nodes. The formula is as follows:


$$\begin{array}{c} S_{i,j}=\text{Euclidea}(E_{i,}E_{j}) \end{array}$$ (18)

Among them, Euclidea(a,b) represents calculating the Euclidean distance between vectors a and b. $S\in R^{N\times N}$ is the similarity matrix containing all nodes. Then, S is multiplied by the original adjacency matrix $A\in R^{N\times N}$ to obtain the weighted similarity matrix $S^{w}\in R^{N\times N}$.

Next, based on $S^{w}$, calculate the aggregation degree of the target node for different classes of adjacent nodes in the graph structure. The formula is as follows:


$$\begin{array}{c} \overset{i}{\underset{\text{x},\text{y}}{F}}=\frac{\sum_{j=\text{1}}^{N_{\text{y}}}\overset{w}{\underset{i,j}{S}}}{\sum_{j=\text{1}}^{N_{\text{y}}}A_{i,j}}(i=\text{1},\text{2},\cdot \cdot \cdot ,N_{\text{x}}) \end{array}$$ (19)

In the above formula, the numerator calculates the total weighted similarity between node i of class x and adjacent nodes of class y (x,y∈{tr,addr}); the denominator calculates the number of adjacent nodes of class y for node i of class x in the graph structure. The aggregation degree value $\overset{i}{\underset{\text{x},\text{y}}{F}}$ is obtained by dividing the two. The higher its value, the closer the relationship with other nodes.

Then, based on $\overset{i}{\underset{\text{x},\text{y}}{F}}$, the mean values of affinity for multi-class normal/abnormal nodes, $\overset{\text{n}}{\underset{\text{x},\text{y}}{\gamma }}$ and $\overset{\text{a}}{\underset{\text{x},\text{y}}{\gamma }}$, are calculated. The formula is as follows:


$$\begin{array}{c} \overset{\text{z}}{\underset{\text{x},\text{y}}{\gamma }}=\frac{\text{1}}{\overset{\text{z}}{\underset{\text{x}}{N}}}\sum_{i\in \overset{\text{z}}{\underset{\text{x}}{I}}}\overset{i}{\underset{\text{x},\text{y}}{F}}(\text{z}\in \{\text{n},\text{a}\}) \end{array}$$ (20)

where, when z=n, the function calculates the affinity mean between normal nodes and $\overset{\text{z}}{\underset{\text{x}}{N}}$ is the number of normal nodes of class x, $\overset{\text{z}}{\underset{\text{x}}{I}}$ is the normal node index set of class x; similarly, when z=a, the function calculates the affinity mean of abnormal nodes. The two means reflect the average level of the respective degree of aggregation of the normal and abnormal node sets, respectively.

Finally, based on $\overset{\text{n}}{\underset{\text{x},\text{y}}{\gamma }}$ and $\overset{\text{a}}{\underset{\text{x},\text{y}}{\gamma }}$, the adjacency adaptation loss function $\overset{t}{\underset{\text{AA}}{L}}$ is derived from the following equation:


$$\begin{array}{c} \overset{t}{\underset{\text{AA}}{L}}=\text{max}(\text{0},C-\left(\overset{\text{n}}{\underset{\text{x},\text{y}}{\gamma }}-\overset{\text{a}}{\underset{\text{x},\text{y}}{\gamma }}\right)) \end{array}$$ (21)

where the predefined parameter C is subtracted by the overall adjacency aggregation degree difference $\left(\overset{\text{n}}{\underset{\text{x},\text{y}}{\gamma }}-\overset{\text{a}}{\underset{\text{x},\text{y}}{\gamma }}\right)$ to obtain an intermediate result, which reflects the deviation between the positive – negative aggregation degree difference of the pseudo-abnormal nodes generated by BF-HGN and the expected difference.

#### 3.6.2 Adaptive feature space regulation loss function.

Since the AA loss function only considers the adjacency aggregation degree during the generation process of pseudo-anomalous nodes and fails to take into account the connection of the feature space distribution among node sets. Therefore, combining the idea that the feature distribution of hard-anomalous node sets is more conducive to model learning, we design the Adaptive Feature Space Regulation (AFSR) Loss Function. It focuses on modeling the anomalous node sets that are highly similar to the normal node set in the feature space, so as to enhance the model’s ability to learn complex decision boundaries and enable it to effectively capture the key feature differences at class boundaries during the training process. The formula of the AFSR loss function is as follows:


$$\begin{array}{c} \overset{\text{x},t}{\underset{\text{AFSR}}{L}}=\frac{\text{1}}{\overset{\text{a}}{\underset{\text{x}}{N}}}\sum_{i=\text{1}}^{\overset{\text{a}}{\underset{\text{x}}{N}}}\sqrt{\sum_{j=\text{1}}^{d}(\overset{{\text{a}}^{\prime \prime }}{\underset{\text{x},ij}{H}}-\overset{{\text{a}}^{\prime }}{\underset{\text{x},ij}{H}}{)}^{\text{2}}} \end{array}$$ (22)

To analyze the above equation: the innermost $\sum_{j=\text{1}}^{d}(\overset{{\text{a}}^{\prime \prime }}{\underset{\text{x},ij}{H}}-\overset{{\text{a}}^{\prime }}{\underset{\text{x},ij}{H}}{)}^{\text{2}}$, where $\overset{a^{\prime \prime }}{\underset{x,ij}{H}}$ is the eigenvalue of the generated pseudo-abnormal nodes in the ith sample, jth embedding vector dimension, and $\overset{{\text{a}}^{\prime }}{\underset{\text{x},ij}{H}}$ is the eigenvalue of the corresponding position of the noisy normal node. This part calculates the squared value of the difference between ${\overset{\text{a}}{\underset{ij}{E}}}^{\prime \prime }\;$and $\overset{\text{o}}{\underset{ij}{E}}$, which can amplify the feature difference, so that the subtle difference can also be significantly reflected in the subsequent calculation. The intermediate layer $\sqrt{\sum_{j=\text{1}}^{d}(\overset{{\text{a}}^{\prime \prime }}{\underset{\text{x},ij}{H}}-\overset{{\text{a}}^{\prime }}{\underset{\text{x},ij}{H}}{)}^{\text{2}}}$ takes the square root of the difference value, and takes into account the differences of the characteristics of each dimension, and obtains the value reflecting the degree of the overall characteristic differences of the sample. Finally, the function sums up the difference values of all the nodes and divides them by the number of nodes $\overset{\text{a}}{\underset{\text{x}}{N}}$, and calculates the average AFSR loss.

#### 3.6.3 Overall loss function.

On the basis of the AA and AFSR loss function, we construct a complete categorization loss system by introducing the BCE loss function [53]. The formula for the BCE loss is as follows:


$$\begin{array}{c} \overset{\text{x},t}{\underset{\text{BCE}}{L}}=\sum_{i=\text{1}}^{N_{a}+N_{b}}w_{\text{1}}\overset{\text{x},t}{\underset{i}{y}}log(\overset{\text{x},t}{\underset{i}{P}})+w_{\text{0}}(\text{1}-\overset{\text{x},t}{\underset{i}{y}})log(\text{1}-\overset{\text{x},t}{\underset{i}{P}}) \end{array}$$ (23)

where $\overset{\text{x},t}{\underset{i}{P}}$denotes the probability that the node output by the classifier is a normal node; $\overset{\text{x},t}{\underset{i}{y}}$ is the label of node i, with a value of 1 if the node is labeled as a normal node and 0 otherwise; and $w_{\text{1}}$ and $w_{\text{0}}$ are the category weights.

Finally, the hyperparameter ?? performs a weighted summation of the three loss functions to adjust the influence of different losses on the BF-HGN:


$$\begin{array}{c} L=\sum_{\text{x}\in \text{X}}\sum_{t\in T}\overset{\text{x},t}{\underset{\text{BCE}}{L}}+\text{l}\cdot \overset{t}{\underset{\text{AA}}{L}}+(\text{1}-l)\overset{\text{x},t}{\underset{\text{AFSR}}{L}} \end{array}$$ (24)

where X={tr,addr} and T is the set of time series

## 4 Experiments

### 4.1 Dataset

Elliptic++ is currently the largest publicly available heterogeneous dataset of Bitcoin transactions [13,14], containing two types of nodes, transaction (Tr) and address (addr), as well as four types of edge relationships: Tr-Tr, addr-addr, Tr-addr, and addr-Tr. The dataset is divided into 49 time points by time, where 1–30 is the training set, 31–35 is the validation set, and 36–49 is the test set. To meet the research needs, two preprocesses are performed on the dataset: one is to divide the edge relations by time points to clarify the temporal correlation; In addition, the labeled abnormal samples are relabeled as the unknown class.

**Fig 5:**
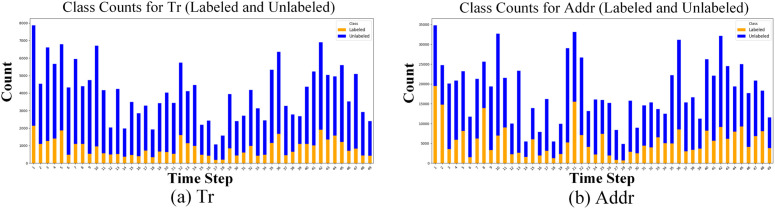
Number of nodes of each type under each point in time.

**Fig 6:**
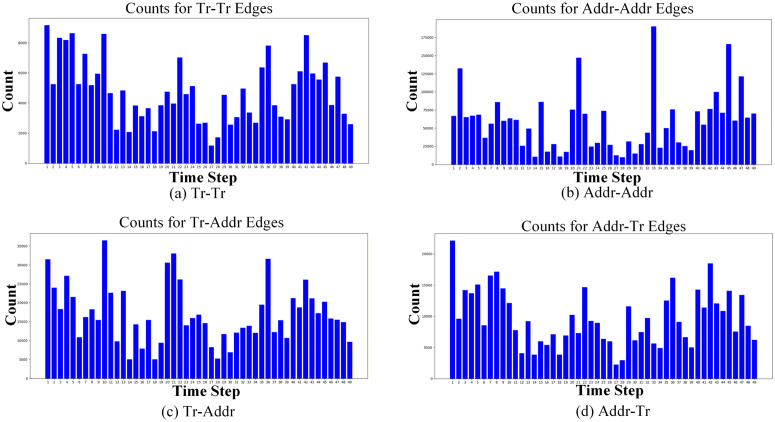
Number of edges of each type under each point in time.

The node data is shown in Fig 5 The Tr node contains 167-dimensional features (including time information), with a total of 203,769 nodes, of which 42,019 are positive samples of legitimate transactions; the addr node contains 56-dimensional features (including time information), with a total of 920,691 nodes, of which 338,871 are positive samples of legitimate transactions, and the rest of the nodes are labeled as unknown. Edge data as shown in Fig 6 has four types including Tr-Tr (234,355), Addr-Addr (2,868,964), Tr-Addr (837,124) and Addr-Tr (477,117), and the edge relationship is featureless.

### 4.2 Evaluation protocol

The binary confusion matrix is the core framework for quantifying the error between the true value and the prediction result, and contains three key metrics: precision, recall, and F1 value.

Precision is calculated by the formula:


$$\begin{array}{c} precision=\frac{TP}{TP+FP} \end{array}$$ (25)

where TP is the number of positive cases correctly identified and FP is the number of negative cases misjudged as positive cases, this indicator reflects the accuracy of the model in determining positive cases. Recall is calculated as:


$$\begin{array}{c} ecall=\frac{TP}{TP+FN} \end{array}$$ (26)

where FN is the number of missed positive cases, which measures the model’s ability to capture actual positive cases.

The F1 value is used as a reconciled average of the two and is calculated as:


$$\begin{array}{c} F\text{1}=\text{2}\times \frac{precision\times recall}{precision+recall} \end{array}$$ (27)

This metric comprehensively evaluates the overall performance of the model in the classification task by balancing precision and recall.

### 4.3 Implementation

This experiment is based on the PyTorch framework and uses NVIDIA RTR 3090 GPUs to accelerate model training and inference. The optimizer is selected from Adam [54], and the initial learning rate is set to 0.001 to balance the training stability and convergence speed. For data sampling, subgraphs from five consecutive time points are randomly selected as training samples each time to enhance the model’s generalization ability. The training configuration is 100 epochs to ensure that the data features are fully captured. For the class imbalance problem, a binary cross-entropy loss function is applied, and the class weights $w_{\text{1}}$ and $w_{\text{0}}$ are set to 0.35 and 0.65, respectively, to optimize the recognition of a few classes. In addition, the variance σ and mean μ in Eq. (23) are 0.005 and 0.015, respectively. The neighbor aggregation difference C in Eq. (21) is 0.7.

### 4.4 Comparison with state-of-the-art methods

In this paper, we propose a novel baseline method in the field of Bitcoin transaction anomaly detection and conduct comparison experiments with existing mainstream homogeneous graph static/dynamic semi-supervised and unsupervised methods. Table 1 presents the experimental comparison results of the BF-HGN with multiple baseline methods on the Elliptic++ dataset. To guarantee the reliability of the results, each method repeats the experiment 10 times and takes the average value as the evaluation index. The analysis shows that BF-HGN outperforms the comparison methods.

**Table 1:** Comparative experiments between the method of this paper and various current state-of-the-art methods.

Learning type	Method	Tr			Addr		
		F1	Pre	Re	F1	Pre	Re
Static semi-supervised	GCN	0.4195	0.5102	0.3562	0.4777	0.4692	0.4859
	Skip-GCN	0.4304	0.5227	0.3659	0.4860	0.4580	0.5176
Static unsupervised	AEGIS	0.3873	0.4376	0.3472	0.4341	0.3852	0.4974
	TAM	0.5121	0.5409	0.4852	0.4857	0.4362	0.5479
Static semi-supervised (Hete-Graph)	HAN	0.4901	0.5581	0.4368	0.5290	0.5103	0.5492
	HGT	0.4411	0.4776	0.4097	0.4315	0.4339	0.4291
	HetGNN	0.5464	0.5734	0.5218	0.5520	0.5215	0.5864
Dynamic unsupervised	GADY	0.5274	0.5942	0.4739	0.5044	0.4778	0.5342
Dynamic semi-supervised	EvolveGCN-H	0.4272	0.5214	0.3618	0.4420	0.4524	0.4319
	EvolveGCN-O	0.5240	0.6217	0.4528	0.4726	0.4281	0.5269
	GPN	0.6050	0.7018	0.5317	0.5567	0.5139	0.6074
Dynamic semi-supervised (Hete-Graph)	BF-HGN (&FC)	0.6184	0.7131	0.5452	0.5743	0.5296	0.6236
	BF-HGN (&FE)	0.6220	0.7249	0.5392	0.5707	0.5284	0.6193
	BF-HGN	**0.6301**	**0.7265**	**0.5563**	**0.5784**	**0.5367**	**0.6261**

First, in comparison with static semi-supervised methods GCN [15] and Skip-GCN [55], BF-HGN achieves +19.97%/ + 8.24%@F1 on Tr and Addr node sets, respectively. This result verifies the significant role of temporal information embedding in node features and the introduction of heterogeneous information across the node classes in the optimization of the model. Secondly, static unsupervised learning methods are AEGIS [56] and TAM [38], BF-HGN achieves +11.8%/ + 8.27%@F1 on the Tr and Addr node sets, respectively, when compared with the better-performing TAM. While comparing with the dynamic unsupervised method GADY [57], BF-HGN also has + 10.27%/ + 7.4%@F1, indicating that training with partially labeled normal nodes enhances model targeting and improves performance and generalization. Finally, in the dynamic semi-supervised task, the GPN [58] optimized based on EvolveGCN-O[17] is suitable for the Bitcoin anomaly detection task, while BF-HGN still achieves +2.49%/ + 2.17%@F1 on the Tr and Addr node sets. This confirms that the embedding of heterogeneous information between node classes can enrich the model input and enhance the feature expression capability.

Furthermore, to fully verify the superiority of BF-HGN in heterogeneous graph modeling, we compare BF-HGN with three heterogeneous graph methods: HAN [59], HGT [60], and HetGNN [61]. Experimental results show that BF-HGN outperforms HAN by +14%/ + 4.94%@F1 on the Tr and Addr node sets, HGT by +18.9%/ + 14.69%@F1, and HetGNN by +8.37%/ + 2.64%@F1. This demonstrates that the bidirectional fusion and heterogeneous feature extraction mechanism of BF-HGN is more suitable for dynamic Bitcoin transaction graphs than general heterogeneous graph models.

Additionally, experiments are conducted in this section for comparison of three feature transformation/expansion methods (BF-HGN (& FC), BF-HGN (& FE), and BF-HGN) proposed in Section 2.1. These methods achieve 61.84%/62.2%/63.01%@F1 on the Tr node set, and 57.43%/57.07%/57.84%@F1 on the Addr node set, respectively. These results demonstrate the optimality of the joint feature extraction strategy of Bi-EvolveGCN and Bi-EvolveRGCN, verifying the pivotal role of node class relationship information embedding in the feature extraction stage.

Overall, the experimental results show that BF-HGN performs better on the Tr node set than on the Addr node set. When combined with the dataset feature scores, it is evident that the Tr node set has a smaller data size and a lower proportion of abnormal nodes. This suggests that the method has a significant advantage when dealing with small samples and datasets with a low proportion of abnormalities.

In summary, BF-HGN has two unique advantages. First, it provides richer semantic information for single-category node set classification by effectively mining cross-node class associative information. Second, BF-HGN balances the sample classes during pseudo-abnormal node sample generation. This significantly improves anomaly detection performance.

### 4.5 Ablation experiment

To clearly verify the effectiveness of BF-HGN and the impact of each innovative module on performance, this section focuses on the core innovative directions proposed by the research. Ablation expriments are conducted on basic innovation modules, such as Bi-EvolveGCN, Bi-EvolveRGCN, CC, AA loss function and AFSR loss function.

The following methods are validated sequentially in this study: 1) Base (EvolveGCN + EvolveRGCN + MLP); 2) Base + Bi-EvolveGCN (Bi-EvolveGCN + EvloveRGCN + MLP); 3) Base + Bi-EvolveRGCN (EvolveGCN-O + Bi-EvolveRGCN + MLP); 4) Base + MFFE (Bi-EvolveGCN-O + Bi-EvloveRGCN + MLP). 5) Base + $L_{\text{AA}}$ (Base embeds a class-balanced classifier and removes the loss function $L_{\text{AFSR}}$); 6) Base + $L_{\text{AFSR}}$(Base embeds a class-balanced classifier and removes the loss function $L_{\text{AA}}$); and 7) Base + CC (Base embeds a class-balanced classifier and two loss functions). 8) BF-HGN. As shown in Table 2, BF-HGN achieves the following on the Tr and Addr datasets, respectively, as compared to the baseline method: + 10.61%/ + 10.58%@F1, + 10.48%/ + 10.86%@Pre, and +10.35%/ + 9.92%@Re.

**Table 2:** Ablation experiments for each innovative point of the method described in this paper.

Datesets Method	Tr			Addr		
	F1	Pre	Re	F1	Pre	Re
Base	0.5240	0.6217	0.4528	0.4726	0.4281	0.5269
Base+Bi-EvolveGCN	0.5359	0.6334	0.4639	0.4835	0.4375	0.5402
Base+Bi-EvolveRGCN	0.5337	0.6368	0.4593	0.4891	0.4483	0.5377
Base+ MFFE	0.5646	0.6507	0.4985	0.5112	0.4693	0.5608
Base+$L_{\text{AA}}$	0.5551	0.6491	0.4821	0.5006	0.4562	0.5547
Base+$L_{\text{AFSR}}$	0.5736	0.6843	0.4937	0.5216	0.4827	0.5719
Base+CC	0.5962	0.7016	0.5183	0.5533	0.5113	0.6029
Our model	**0.6301**	**0.7265**	**0.5563**	**0.5784**	**0.5367**	**0.6261**

Next, the differences among 2), 3), and 4) are primarily reflected in the feature extraction module. Compared to the Base model on the Tr and Addr node sets, 2) achieves + 1.19%/ + 1.09% @F1, 3) achieves + 0.97%/ + 1.65% @F1, and4) achieves +4.06%/ + 3.86% @F1. The experimental results demonstrate the significant advantages of the bidirectional temporal feature embedding method over the unidirectional mechanism. This method uses a fusion embedding of forward and reverse temporal information to capture contextual dependencies more efficiently.

**Fig 7:**
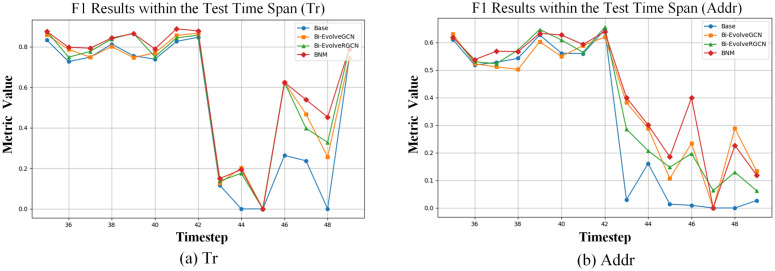
F1 results within time span 35-49.

Additionally, to explore the model’s ability to capture long-time-distance dependencies, this section selects the subgraphs of time points 35–49 for experimental comparison, and the results are shown in Fig 7 Analyzing the F1 as the core index reveals that each method performs well in the time interval 35–42. Since time point 43, model performance fluctuates. Further analysis of the experimental data reveals that the bidirectional temporal embedding methods (2), 3), 4) and 8)) significantly outperform Base in capturing long-range temporal dependencies.

Next, 5), 6), and 7) differ mainly in the classification phase and all show significant optimization compared to the base. On the Tr and Addr node sets, 5) achieves + 3.11%/ + 2.8%@F1, 6) achieves 4.96%/ + 4.9% @F1, and 7) achieves +7.22%/ + 8.07%@F1. Further analysis shows the following: first, CC balances samples by class by generating discrete abnormal nodes. This mechanism enables the model to learn feature representations from richer class distributions. It avoids a single class from dominating the training process, thus improving the model’s ability to capture multi-class patterns. Second, AA loss function plays a key role in optimizing the distribution of pseudo-abnormal nodes. It integrates the distance difference between real abnormal samples and neighboring nodes, adjusting the generated pseudo-abnormal node neighbor aggregation features. This enhances the model’s ability to discriminate neighboring anomalous node patterns. Lastly, AFSR loss function aligns the feature boundaries of the pseudo-abnormal and hard abnormal sample classes through spatial alignment, prompting the model to learn the complex anomaly class boundaries.

Finally, to further validate that the bidirectional fusion module can effectively capture the long-range temporal dependencies typically overlooked by unidirectional models, we perform a node-level case study in this section. Specifically, we randomly select one Tr node (ID: 54771037) and one Addr node (ID: 1Ej…isz) at time step 35, and statistically analyze the cross-timestep anomaly correlation patterns of these two nodes under both the Base model and BF-HGN. The detailed statistical results are summarized in Table 3, where the column Truth denotes the ground-truth number of associated anomalous nodes.

**Table 3:** Statistical results of cross-timestep anomaly correlations for sampled nodes at time step 35 under Base and BF-HGN.

Time Target		36	37	38	39	40	41	42	43	44	45	46	47	48	49
Tr(54771037)	Truth	3	0	4	5	2	7	2	5	6	4	12	8	4	3
	Base	2	0	2	4	1	7	0	3	3	2	2	0	0	3
	BF-HGN	2	0	2	5	1	7	0	4	5	3	9	5	4	3
Addr(1Ej…isz)	Truth	9	6	8	15	7	18	4	7	15	7	25	24	6	2
	Base	7	6	4	9	5	18	2	3	12	2	6	11	1	0
	BF-HGN	7	6	4	15	4	18	2	5	15	4	19	20	5	1

Between time steps 36 and 42, the anomaly detection performance of the Base model and BF-HGN shows little difference. However, after time step 42, the number of accurately detected anomalous nodes for both Tr and Addr node categories under BF-HGN is almost consistently higher than that under the Base model. In particular, at time step 46, BF-HGN detects 13 more anomalous Addr nodes associated with 1Ej…isz than the Base model. These results clearly indicate that BF-HGN exhibits superior long-range anomaly detection capability compared with the Base method.

It can be observed that the anomaly detection performance of BF-HGN presents an overall upward trend after time step 35, which quantitatively verifies that the proposed bidirectional fusion model has significantly stronger long-range anomaly mining ability than the unidirectional Base model.

### 4.6 Computational complexity analysis

Since the bidirectional temporal fusion mechanism requires forward and backward propagation, it may potentially double the computational cost. To clarify the actual computational overhead of our model, we perform a systematic time and space complexity analysis of BF-HGN and compare it with the baseline method in this section.

First, we analyze the complexity of the baseline model EvolveGCN-O. For a dynamic graph with T time steps, N transaction nodes, M address nodes and d-dimensional node features, the time complexity of EvolveGCN-O is $\text{O}(T({Nd}^{\text{2}}+{Md}^{\text{2}}))$, and its space complexity is O((N+M)d), which is used to store node features and model parameters.

For the proposed BF-HGN, its bidirectional temporal fusion module consists of Bi-EvolveGCN and Bi-EvolveRGCN. The time complexity of Bi-EvolveGCN is $\text{O}(T{Nd}^{\text{2}})$ and the time complexity of Bi-EvolveRGCN is $\text{O}(T{Md}^{\text{2}})$. Benefiting from the parameter sharing mechanism in the forward and reverse temporal update processes, the overall time complexity of the bidirectional fusion stage is $\text{O}(T({Nd}^{\text{2}}+{Md}^{\text{2}}))$, which only increases by a constant factor (less than twice) compared with the baseline. Meanwhile, the space complexity of BF-HGN remains O((N+M)d), which is completely consistent with that of the baseline model.

Furthermore, the feature dimensions of the Elliptic++ dataset are 56 and 167, and the number of neural network layers in the feature extraction stage is only 4. This shallow network structure results in an extremely small number of model parameters and low memory usage. In the context of large-scale computing power support, this extra memory overhead is almost negligible.

In summary, compared with the baseline EvolveGCN-O, BF-HGN only introduces a small amount of additional computational overhead, but achieves significant performance improvement in Bitcoin transaction anomaly detection. The proposed model strikes a desirable balance between computational efficiency and detection performance.

### 4.7 Quantitative experiment

**Fig 8:**
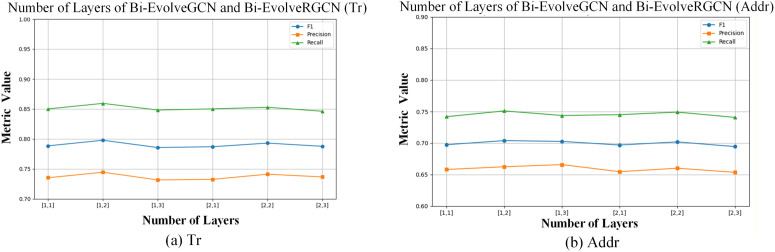
Quantitative experiments on the number of layers of Bi-EvolveGCN and Bi-EvolveRGCN.

**Fig 9:**
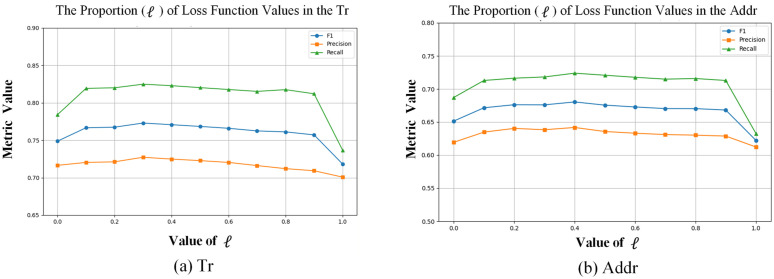
Quantitative experiment on the proportional value of the loss functionℓ.

**Fig 10:**
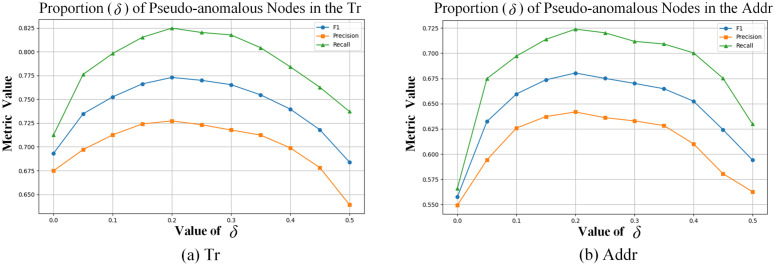
Experimental comparison of the pseudo-abnormal sample proportionδ.

In this section, quantitative experiments are conducted to optimize the key hyperparameters of BF-HGN, aiming to determine the optimal parameter configuration. Figs 8–10 present the experimental results of the three core hyperparameters, with detailed analyses carried out in sequence.

First, we focus on the layer number combination [x, y] of Bi-EvolveGCN and Bi-EvolveRGCN. The experiment evaluates the optimal parameters by limiting the combination within the range of [1, 3]. As shown in Fig 8, BF-HGN achieves the best performance when the number of layers in Bi-EvolveGCN is 1 and that in Bi-EvolveRGCN is 2. Specifically, the layer combination [1, 1] leads to the loss of effective information during classification due to insufficient feature extraction; whereas the configuration [1, 2] elicits the optimal synergistic effect between feature extraction and the classifier, enabling the model to exhibit excellent performance in node detection tasks. When the layer combination exceeds this configuration (e.g., [2, 1] or [3, 2]), BF-HGN tends to suffer from overfitting, resulting in a decline in generalization ability and ultimately affecting the detection performance.

During model training, we systematically tune the scaling parameter 𝓁 of the loss function in Eq. (24) to achieve balanced optimization of multiple loss terms. The experimental results are shown in Fig 9. In experiments on the Tr node set, model performance peaks at 𝓁=0.3, which can effectively balance the optimization objectives of each loss term. When 𝓁 deviates from the optimal value, regardless of whether it increases or decreases, the model performance shows a downward trend. This is due to an excessively high weight of a single loss term leads to an imbalance in the optimization process, suppressing the effective adjustment of model parameters by other loss terms. In experiments with the Addr node set, the model achieves optimal performance at ℓ=0.3/0.4. Based on the above experimental results, ℓ=0.3 is finally selected as the final parameter.

Finally, we systematically analyzed the influence of the proportion δ of pseudo – anomalous samples mentioned in Section 3.2 on BF-HGN. As shown in Fig 10, on the Tr node set, when δ=0.2, the model performance reaches the optimum, and both the F1 value and key evaluation indicators show peak values; as δ continues to increase, the detection accuracy shows a downward trend, so δ=0.2 is determined as the optimal value. On the Addr node set, the best detection effect is also achieved whenδ=0.2, and the recognition ability of BF-HGN for normal/anomalous samples reaches the optimal balance. Therefore, δ=0.2 is finally set.

### 4.8 Visualization experiment

To deeply explore the performance of pseudo-abnormal nodes generated by BF-HGN, the subgraph of time 5 is selected as the object of visualization and analysis in this experiment, and the t-SNE dimensionality reduction algorithm is used to realize the visual presentation of the feature space. By comparing the benchmark method and the BF-HGN method on the Tr node set and Addr node set in Fig 10, the normal nodes are marked by blue circles, green circles mark the pseudo-abnormal nodes generated by BF-HGN, and green dashed lines frame the distribution area of pseudo-abnormal nodes.

**Fig 11:**
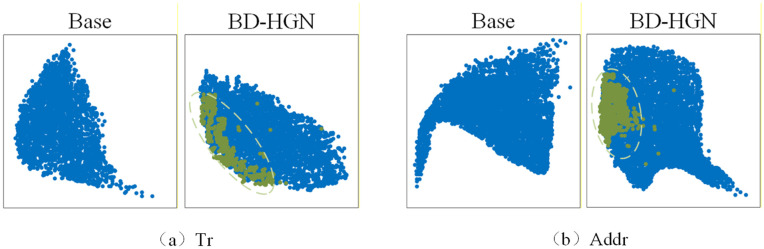
Visualization experiments of BF-HGN with benchmark methods.

As shown in Fig 11a, the normal nodes in the base method form a clear, aggregated boundary in their spatial distribution. The node clusters generated by BF-HGN, however, are concentrated in a specific spatial region at the lower-left boundary position of the feature space, where the pseudo-abnormal nodes are mainly located. Though there is significant overlap between the distributions of pseudo-abnormal and normal nodes, a subtle spatial mismatch between the two can be observed upon closer inspection. The node distributions presented by both the base method and BF-HGN have significant boundary features, as shown in Fig 11b. The difference is that the pseudo-abnormal nodes generated by BF-HGN are clustered toward the left boundary of the space. This cluster does not completely overlap with the normal node set in terms of spatial dimension. This forms a unique distribution difference.

The visualization results show that the pseudo-abnormal nodes generated by BF-HGN are highly concealed and difficult to mine due to their spatial distribution characteristics, which are similar to but different from those of normal nodes. These characteristics provide more challenging training samples for the anomaly detection model and can effectively enhance its ability to recognize and detect anomalies in complex scenarios.

## 5 Conclusion and outlook

### 5.1 Conclusion

In this paper, we carry out systematic research on bitcoin transaction anomaly detection task and propose several innovative methods: firstly, to meet the practical requirements, we define the dynamic heterogeneous graph semi-supervised bitcoin anomaly detection task and design the Bi-directional Fusion Heterogeneous Graph Network (BF-HGN) to construct the basic framework. Second, in feature extraction, we improve upon RGCN to construct EvolveRGCN and combines EvolveGCN to design a gradual scheme. It also introduces LSTM to capture temporal features and deeply mines dynamic features through a fusion strategy. Further, we propose the Multi-type Feature Fusion Extractor. This improves the dynamic relationship modeling capability by capturing the upper and lower time-point subgraph associations. Lastly, we address the class imbalance problem caused by unlabeled anomalous samples by designing Class-balanced Classifiers. These classifiers balance the training data class distribution by generating pseudo-abnormal nodes constrained by AA and AFSR loss function.

### 5.2 Outlook

Future research can be extended to a broader range of financial transaction scenarios, thereby strengthening risk prevention and control capabilities. Further exploration of the optimization space of feature extraction and fusion strategies reveals potential associations in complex data and injects richer semantic information into the model. Meanwhile, continuous efforts should be made to refine the optimization path of loss functions to improve the generation quality of pseudo-anomalous nodes, so as to promote the security and stability of anomaly detection technologies in Bitcoin transactions and related fields. In addition to technical advancements, future studies should incorporate regulatory, ethical, and societal considerations into the design of anomaly detection systems. Inspired by the sociotechnical framework proposed by Rahman et al. [62], responsible and trustworthy FinTech development can be better supported in blockchain transaction surveillance, particularly with respect to regulatory compliance, transparency, and social accountability.
